# Defining a clinically meaningful within-patient change threshold for the Cohen-Mansfield Agitation Inventory in Alzheimer’s dementia

**DOI:** 10.3389/fneur.2024.1379062

**Published:** 2024-07-23

**Authors:** Juliette Meunier, Kristin Creel, Angély Loubert, Klaus Groes Larsen, Jyoti Aggarwal, Nanco Hefting, Dorothee Oberdhan

**Affiliations:** ^1^Modus Outcomes, Lyon, France; ^2^H. Lundbeck A/S, Valby, Denmark; ^3^Otsuka Pharmaceutical Development & Commercialization Inc., Princeton, NJ, United States

**Keywords:** Alzheimer’s disease, agitation, dementia, behavioral and psychological symptoms of dementia, Cohen-Mansfield Agitation Inventory, meaningful within-patient change, clinical relevance, brexpiprazole

## Abstract

**Introduction:**

The Cohen-Mansfield Agitation Inventory (CMAI) quantifies the frequency of agitation behaviors in elderly persons. This *post hoc* analysis of data from the brexpiprazole clinical program aimed to determine a meaningful within-patient change (MWPC) threshold for CMAI Total score among patients with agitation associated with dementia due to Alzheimer’s disease.

**Methods:**

Data were included from three 12-week, multicenter, randomized, double-blind, placebo-controlled, parallel-arm trials of brexpiprazole for the treatment of agitation associated with dementia due to Alzheimer’s disease (ClinicalTrials.gov identifiers: NCT01862640, NCT01922258, NCT03548584). Change in CMAI Total score (range 29–203; higher scores indicate higher frequency of agitation behaviors) from baseline to Week 12 was the primary endpoint in each trial. MWPC thresholds were estimated from anchor-based mean change analyses and receiver operating characteristic (ROC) curves. The Clinical Global Impression–Severity of illness (CGI-S) and Clinical Global Impression–Improvement (CGI-I) scales, both as related to agitation, were used as anchors. Empirical cumulative distribution functions (eCDFs) and probability density functions (PDFs) were plotted as supportive evidence. Distribution-based methods were also employed.

**Results:**

Data from 898 patients were analyzed (mean age, 73.7 years; mean baseline CMAI Total score, 73.8). The mean CMAI Total score change corresponding to a difference of small improvement vs. stable (CGI-S one-point decrease vs. no change), or minimally improved vs. no change (CGI-I rating of 3 vs. 4), ranged from −10.6 to −13.5 points. The mean CMAI Total score change corresponding to a difference of moderate improvement vs. stable (CGI-S two-point decrease vs. no change), or much improved vs. no change (CGI-I rating of 2 vs. 4), ranged from −20.2 to −25.7 points. ROC curve analyses generally produced smaller estimates of meaningful change. eCDFs and PDFs showed good distribution and separation of CMAI Total score change between CGI-S/CGI-I categories. In distribution-based analyses, the minimal detectable change for CMAI Total score (10.5–11.8 points) was generally lower than anchor-suggested thresholds.

**Conclusion:**

Triangulation of evidence from anchor- and distribution-based analyses supports an MWPC threshold for CMAI Total score of −20 points, with a threshold range of −15 to −25 points, in patients with agitation associated with dementia due to Alzheimer’s disease.

## 1 Introduction

Agitation is a common clinical manifestation of Alzheimer’s dementia, which increases the burden of disease for patients and caregivers (1–3). Agitation associated with dementia is an important target for treatment, both pharmacological and non-pharmacological, provided that no underlying modifiable causes of agitation (such as pain) can be identified (4–6). Caregivers consider any reduction in the frequency of agitation behaviors to be a meaningful improvement, as well as change from physical to verbal aggression, and from aggressive to non-aggressive behaviors (7).

The Cohen-Mansfield Agitation Inventory (CMAI) is a tool for assessing the frequency of agitation behaviors in elderly persons, based on information from the patient’s caregiver (8, 9). The “long-form” version comprises 29 agitation behaviors, the majority of which can be categorized into three distinct factors: aggressive behaviors, physically non-aggressive behaviors, and verbally agitated behaviors (a fourth factor, hiding and hoarding, emerged in some analyses) (9, 10). The CMAI was initially developed and validated for use in nursing homes and has since been adapted for use in community-based settings (8, 9, 11–14). A 14-item “short-form” version of the CMAI has also been developed, in which items from the long form were combined and scored differently, creating a related but distinct tool (15, 16).

Until recently, no medications were approved by the US Food and Drug Administration (FDA) specifically for agitation associated with dementia due to Alzheimer’s disease, and treatment options to control symptoms were limited to off-label use of several drug classes, including antipsychotics, sedatives/hypnotics, anxiolytics, and antidepressants (17). Based on evidence from two 12-week, randomized, double-blind, placebo-controlled trials (supported by a third study) that used the 29-item long-form CMAI as the primary endpoint (18, 19) and a 12-week extension trial (20), the atypical antipsychotic, brexpiprazole, was approved by the FDA in May 2023 for the treatment of agitation associated with dementia due to Alzheimer’s disease (21).

Although the CMAI has been used in various clinical trials in dementia (19, 22), the clinical relevance of changes in CMAI Total score requires further study. There is a need to define a meaningful within-patient change (MWPC) threshold for CMAI Total score in patients with dementia, to indicate the change that corresponds to an important, noticeable improvement in agitation. The aim of this study, a *post hoc* analysis of data from the brexpiprazole clinical program, was to determine a threshold that would constitute an MWPC in CMAI Total score (29-item long form) among patients with agitation associated with dementia due to Alzheimer’s disease. This MWPC was then used to determine *post hoc* clinically meaningful response rates for brexpiprazole vs. placebo.

## 2 Materials and methods

### 2.1 Data sources

This study was a *post hoc* analysis of data from three Phase 3 clinical trials that were originally designed to investigate the efficacy, safety, and tolerability of brexpiprazole for agitation associated with dementia due to Alzheimer’s disease.

#### 2.1.1 Clinical trial design

Trial 1 (ClinicalTrials.gov identifier: NCT01862640), Trial 2 (NCT01922258), and Trial 3 (NCT03548584) were 12-week, multicenter, randomized, double-blind, placebo-controlled, parallel-arm trials, the details of which have been published previously (18, 19). Trial 1 was conducted from July 2013 to March 2017, Trial 2 from October 2013 to March 2017, and Trial 3 from May 2018 to June 2022. The trials were conducted in Europe (including Russia) and North America. Patients were eligible if they met the following criteria: age 55–90 years; diagnosis of probable Alzheimer’s disease (23); Mini Mental State Examination score of 5–22 (24); Neuropsychiatric Inventory (NPI) or NPI – Nursing Home version Agitation/Aggression score of ≥4 (25, 26); and living in a care facility or community-based setting (not living alone). Trial 3 also required patients to meet the International Psychogeriatric Association (IPA) definition of agitation (27), and a CMAI aggressive behavior factor score threshold to ensure that sufficient levels of agitation were present at baseline (10, 19). Eligible patients were randomized to brexpiprazole or placebo for 12 weeks [Trial 1: fixed doses of 1 mg/day, 2 mg/day, or placebo (1:1:1), with an additional 0.5 mg/day arm removed after the trial had started due to new information from other studies indicating that this dose was non-efficacious; Trial 2: flexible doses of 0.5–2 mg/day or placebo (1:1); Trial 3: fixed doses of 2 or 3 mg/day or placebo (2:1)]. Stable background medications for the treatment of Alzheimer’s disease and depression were permitted, whereas antipsychotics, mood stabilizers, anticonvulsants, and benzodiazepines (with some exceptions) were prohibited.

#### 2.1.2 Assessments

The primary endpoint in each trial was the change from baseline to Week 12 in CMAI Total score, calculated as the sum of the 29 CMAI items (“long-form” version; Box 1). Each item is scored on a seven-point scale, where 1 indicates that the patient never shows the behavior and 7 indicates that the behavior occurs several times per hour (based on the previous 2 weeks), giving a total score range from 29 to 203 points (9). In the brexpiprazole trials, the CMAI was completed by the clinician based on an interview with the patient’s caregiver.

BOX 1 **CMAI factors and items (10).**

**Aggressive behaviors**

3. Spitting (including at meals)
4. Cursing or verbal aggression
7. Hitting (including self)
8. Kicking
9. Grabbing onto people
10. Pushing
11. Throwing things
13. Screaming
14. Biting
15. Scratching
21. Hurting self or others
25. Tearing things/destroying property

**Physically non-aggressive behaviors**

1. Pacing, aimless wandering
2. Inappropriate dressing or disrobing
16. Trying to get to a different place
22. Handling things inappropriately
26. Performing repetitious mannerisms
29. General restlessness

**Verbally agitated behaviors**

5. Constant unwarranted requests for attention/help
6. Repetitive sentences or questions
18. Complaining
19. Negativism

**Other behaviors**

12. Making strange noises
17. Intentional falling
20. Eating or drinking inappropriate substances
23. Hiding things
24. Hoarding things
27. Making verbal sexual advances
28. Making physical sexual advances

CMAI, Cohen-Mansfield Agitation Inventory.

Secondary efficacy endpoints included Clinical Global Impression–Severity of illness (CGI-S) as related to agitation, and Clinical Global Impression–Improvement (CGI-I) as related to agitation. The CGI-S is a single-item clinician rating of illness severity, scored on a seven-point scale from 1 (“normal, not at all ill”) to 7 (“among the most extremely ill patients”) (28). The CGI-I is a single-item clinician rating of change relative to baseline, scored on a seven-point scale from 1 (“very much improved”) to 7 (“very much worse”) (28).

### 2.2 Meaningful within-patient change threshold analyses

Analyses to determine an MWPC threshold for CMAI Total score were conducted using methods described in the FDA’s Patient-Focused Drug Development Guidance (29). The analyses were conducted in several stages, using anchor-based methods supplemented by distribution-based methods. Initially, pooled patient data from Trials 1 and 2 were used, then analyses were replicated using data from Trial 3. The rationale for this approach was that Trial 3 was conducted at a later date than Trials 1 and 2, and Trial 3 had additional inclusion criteria to enrich for sufficient agitation at baseline to show a measurable change over time. Finally, the analyses were replicated using pooled data from all three trials.

Analyses were performed independent of treatment arm in the sample of patients who took at least one dose of trial drug (any brexpiprazole dose or placebo) and who had a baseline and at least one post-baseline CMAI Total score measurement. No imputation for missing data was performed. All analyses were performed using SAS version 9.4 (SAS Institute Inc.; Cary, NC, United States).

#### 2.2.1 Anchor-based methods

Anchor-based methods can be used to link changes in the score of interest to external measures, such as clinician global ratings (30). In these analyses, the CGI-S and CGI-I, both as related to agitation, were used as anchor scales. The CGI-S and CGI-I as related to agitation are appropriate anchors because they are straightforward, single-item measures; they have a clear association with the outcome of interest (agitation); and their scores can indicate a clinically meaningful improvement (e.g., a one-point improvement in CGI-S score or a CGI-I score of ≤3) (28). To confirm the suitability of the anchors for determining MWPC thresholds, Spearman rank-order correlations were calculated between change in CMAI Total and factor scores from baseline to Week 12 and (A) change in CGI-S score from baseline to Week 12, and (B) CGI-I score at Week 12. A correlation of ≥0.3 was considered strong enough to warrant the use of the anchor-based methods.

Meaningful within-patient change thresholds were estimated as the mean change in CMAI Total score that corresponded to the following CGI-S comparisons of interest: (A) a small, but clinically observable, improvement (one-point decrease) vs. stable (no change) from baseline to Week 12; and (B) a moderate improvement (two-point decrease) vs. stable (no change) from baseline to Week 12. For the CGI-I, the comparisons of interest were: (A) “minimally improved” (rating of 3) vs. “no change” (rating of 4) at Week 12; and (B) “much improved” (rating of 2) vs. “no change” (rating of 4) at Week 12.

Additionally, MWPC thresholds were estimated from receiver operating characteristic (ROC) curves plotted for change in CMAI Total score from baseline to Week 12 for each anchor. The area under the ROC curve was calculated to determine the ability of the score to discriminate between the two categories of patients defined by the anchor. The MWPC threshold was estimated as the smallest sum of squares of 1- sensitivity and 1- specificity (31).

Empirical cumulative distribution functions (eCDFs) and probability density functions (PDFs) were plotted to depict change from baseline in CMAI Total score according to the change from baseline to Week 12 in CGI-S score, and CGI-I score at Week 12, as supportive evidence for the MWPC thresholds suggested by the mean change and ROC analyses.

#### 2.2.2 Distribution-based methods

Distribution-based methods use statistical parameters related to the distribution of scores in a relevant sample to provide a statistical basis for MWPC threshold selection (30). In this analysis, the following distribution-based parameters for CMAI Total score were calculated: the standard deviation (SD) at baseline (SD_BL_); half SD_BL_ [since a moderate effect size of 0.5 is generally considered clinically significant (32)]; the standard error of measurement (SEM); and the minimal detectable change (MDC). SEM was estimated by multiplying SD_BL_ by √(1 − reliability coefficient), where the reliability coefficient was estimated by the intraclass correlation coefficient (ICC) in stable patients, defined as “no change” on the CGI-I at Week 2. The MDC was calculated as 1.96 × √2 × SEM. These statistics, particularly the MDC, serve to ensure that the thresholds suggested by the anchor-based analyses exceed the measurement error in the scale.

#### 2.2.3 Triangulation

Results of the above analyses were triangulated to determine the MWPC threshold and threshold range (33), primarily considering evidence from the anchor-based mean changes and eCDFs. As FDA guidance favors global severity scales over global change scales as an anchor (due to the possibility of recall bias with global change scales) (29), the CGI-S mean change and eCDF distributions were given more weight than those of the CGI-I.

#### 2.2.4 Responder analyses

Responder analyses were performed using the triangulated MWPC threshold range. The percentage of patients with an improvement in CMAI Total score equal to or greater than the threshold was determined at Week 12, separately for each brexpiprazole and placebo group [except the brexpiprazole 0.5 mg/day arm (*n* = 13)], in each of the three trials.

## 3 Results

### 3.1 Patients

A total of 1,035 patients across the three trials were included (Trial 1, *n* = 424; Trial 2, *n* = 269; Trial 3, *n* = 342). Of these patients, 898 had available data at Week 12 and were included in the MWPC analyses (Trial 1, *n* = 370; Trial 2, *n* = 233; Trial 3, *n* = 295).

Mean age was 73.7 years (range: 51–90 years), 57.0% of patients were female, and 95.5% were White (Table 1). In terms of baseline agitation, mean CMAI Total score was 73.8 points (range 35–140), and the majority of patients were moderately (45.7%) or markedly (43.3%) ill (i.e., CGI-S score of 4 or 5). Further demographic and clinical characteristics by treatment group have been previously published (18, 19).

**Table 1:** Baseline demographic and clinical characteristics.

Characteristic	Trial 1 (*n* = 370)	Trial 2 (*n* = 233)	Trial 3 (*n* = 295)	Trials 1–3 pooled (*n* = 898)
Age, mean (SD), years	73.9 (8.3)	73.2 (8.0)	73.7 (7.6)	73.7 (8.0)
Sex, *n* (%)				
Female	200 (54.1)	148 (63.5)	164 (55.6)	512 (57.0)
Male	170 (45.9)	85 (36.5)	131 (44.4)	386 (43.0)
Race, *n* (%)				
Asian	4 (1.1)	3 (1.3)	3 (1.0)	10 (1.1)
Black or African American	11 (3.0)	7 (3.0)	11 (3.7)	29 (3.2)
White	355 (95.9)	222 (95.3)	281 (95.3)	858 (95.5)
Other	0 (0.0)	1 (0.4)	0 (0.0)	1 (0.1)
Ethnicity, *n* (%)				
Hispanic or Latino	59 (15.9)	15 (6.4)	91 (30.8)	165 (18.4)
Not Hispanic or Latino	309 (83.5)	216 (92.7)	204 (69.2)	729 (81.2)
Unknown	2 (0.5)	2 (0.9)	0 (0.0)	4 (0.4)
CMAI Total score, mean (SD)	71.3 (16.9)	70.1 (16.5)	79.8 (16.2)	73.8 (17.1)
CGI-S as related to agitation, *n* (%)				
Borderline ill (2)	0 (0.0)	0 (0.0)	1 (0.3)	1 (0.1)
Mildly ill (3)	7 (1.9)	10 (4.3)	3 (1.0)	20 (2.2)
Moderately ill (4)	192 (51.9)	116 (49.8)	102 (34.6)	410 (45.7)
Markedly ill (5)	144 (38.9)	85 (36.5)	160 (54.2)	389 (43.3)
Severely ill (6)	27 (7.3)	21 (9.0)	29 (9.8)	77 (8.6)
Among the most extremely ill patients (7)	0 (0.0)	1 (0.4)	0 (0.0)	1 (0.1)

CGI-S, Clinical Global Impression–Severity of illness; CMAI, Cohen-Mansfield Agitation Inventory; SD, standard deviation.

### 3.2 Correlations between CMAI and anchors

Change in CMAI Total score correlated with change in CGI-S score at Week 12 (*r* = 0.61–0.73, depending on the sample) (Table 2). Similar correlations were observed between change in CMAI Total score and CGI-I score at Week 12 (*r* = 0.66–0.73) (Table 2). CMAI factors also correlated with the CGI-S and CGI-I. Thus, the CGI-S and CGI-I were considered to be appropriate anchors for MWPC analyses.

**Table 2:** Spearman correlations between change in CMAI Total and factor scores and anchor scales at Week 12.

	Trials 1 and 2 pooled (*n* = 603)		Trial 3 (*n* = 295)		Trials 1–3 pooled (*n* = 898)	
	CGI-S change	CGI-I	CGI-S change	CGI-I	CGI-S change	CGI-I
CMAI Total	0.61	0.66	0.73	0.73	0.65	0.68
Aggressive behaviors	0.48	0.49	0.61	0.56	0.51	0.51
Physically non-aggressive behaviors	0.58	0.60	0.57	0.58	0.58	0.59
Verbally agitated behaviors	0.42	0.49	0.51	0.57	0.45	0.52

CGI-I, Clinical Global Impression–Improvement; CGI-S, Clinical Global Impression–Severity of illness; CMAI, Cohen-Mansfield Agitation Inventory.

### 3.3 Anchor-based analyses

Considering pooled data from all three trials, using change in CGI-S score as the anchor, patients in the moderate improvement group (*n* = 247) experienced a mean CMAI Total score change of −28.1 points (SD, 14.9), and those in the small improvement group (*n* = 315) experienced a mean change of −19.0 points (SD, 12.5). Patients in the stable group (*n* = 238) experienced a mean CMAI Total score change of −6.1 points (SD, 10.4). Using CGI-I score as the anchor, patients in the much improved group (*n* = 373) experienced a mean CMAI Total score change of −25.8 points (SD, 13.5) and those in the minimally improved group (*n* = 266) experienced a mean change of −15.3 points (SD, 12.3), compared to a mean change of −3.6 points (SD, 8.1) in the no change group (*n* = 120). In all groups, mean CMAI Total score changes were slightly larger in Trial 3 than in Trials 1 and 2 (pooled) (data not shown).

For the comparisons of interest, the mean change in CMAI Total score that corresponded to the difference of small improvement vs. stable (one-point decrease vs. no change) based on CGI-S score, or minimally improved vs. no change (rating of 3 vs. 4) based on CGI-I score, ranged from −10.6 to −13.5 points, depending on the dataset (Table 3). The mean change in CMAI Total score that corresponded to the difference of a moderate improvement vs. stable (two-point decrease vs. no change) based on CGI-S score, or much improved vs. no change (rating of 2 vs. 4) based on CGI-I score, ranged from −20.2 to −25.7 points, depending on the dataset (Table 3).

**Table 3:** Thresholds for MWPC of CMAI Total score obtained using anchor-based mean change and ROC curve analyses.

Trial	CMAI Total score change			
	CGI-S as anchor		CGI-I as anchor	
	Small improvement (one-point decrease) vs. stable (no change)	Moderate improvement (two-point decrease) vs. stable (no change)	Minimally improved (score of 3) vs. no change (score of 4)	Much improved (score of 2) vs. no change (score of 4)
Means analysis				
Trials 1 and 2 pooled	−12.6	−20.2	−12.3	−21.5
Trial 3	−13.5	−25.7	−10.6	−23.6
Trials 1–3 pooled	−12.9	−22.0	−11.7	−22.2
ROC curve analysis				
Trials 1 and 2 pooled	−9	−14	−8	−11
Trial 3	−14	−18	−8	−16
Trials 1–3 pooled	−12	−16	−8	−12

CGI-I, Clinical Global Impression–Improvement; CGI-S, Clinical Global Impression–Severity of illness; CMAI, Cohen-Mansfield Agitation Inventory; MWPC, meaningful within-patient change; ROC, receiver operating characteristic.

In ROC curve analyses, the change in CMAI Total score that maximized the separation between participants with small improvement in CGI-S score and those who were stable ranged from −9 to −14 points and was highest in Trial 3 (Table 3). The corresponding analysis for minimally improved vs. no change in CGI-I score produced more consistent results (−8 points in all datasets). The change in CMAI Total score that maximized the separation between participants with moderate improvement in CGI-S score and those who were stable ranged from −14 to −18 points, and for much improved vs. no change in CGI-I score ranged from −11 to −16 points (Table 3).

### 3.4 Empirical cumulative distribution functions and probability density functions

**Figure 1:**
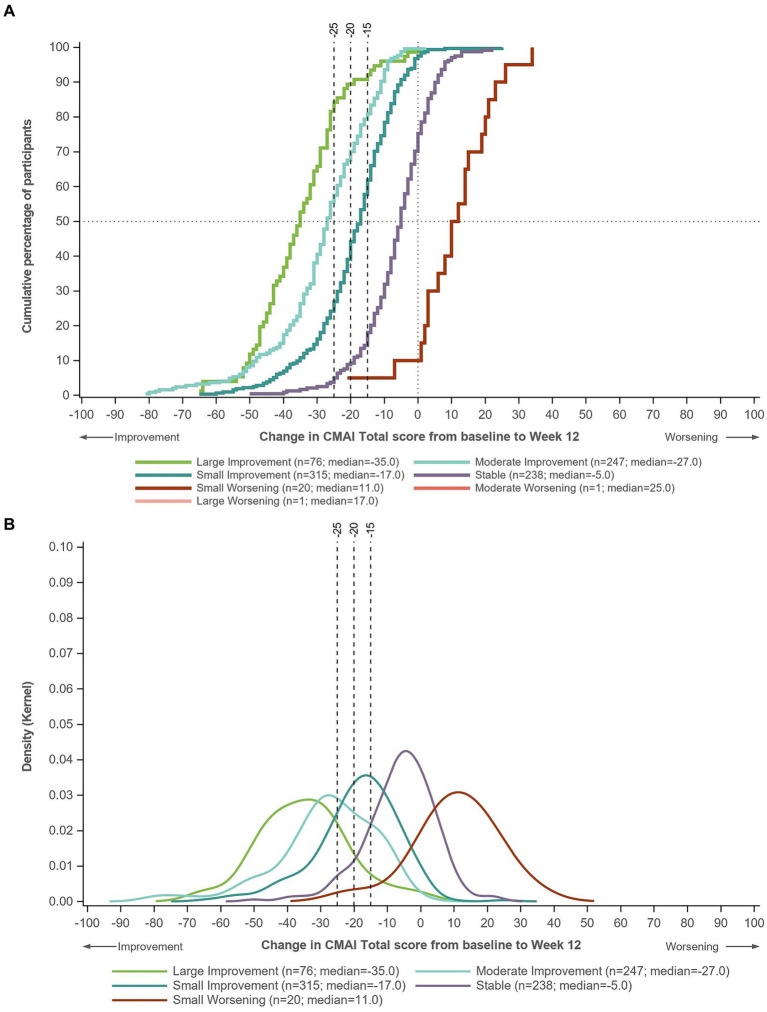
**(A)** eCDF and **(B)** PDF of change in CMAI Total score in groups defined by change in CGI-S score from baseline to Week 12. Data for Trials 1–3 pooled. Large improvement, ≥3-point decrease in CGI-S; moderate improvement, two-point decrease in CGI-S; small improvement, one-point decrease in CGI-S; stable, no change in CGI-S; small worsening, one-point increase in CGI-S; moderate worsening, two-point increase in CGI-S; large worsening, ≥3-point increase in CGI-S. CGI-S, Clinical Global Impression–Severity of illness; CMAI, Cohen-Mansfield Agitation Inventory; eCDF, empirical cumulative distribution function; PDF, probability density function.

**Figure 2:**
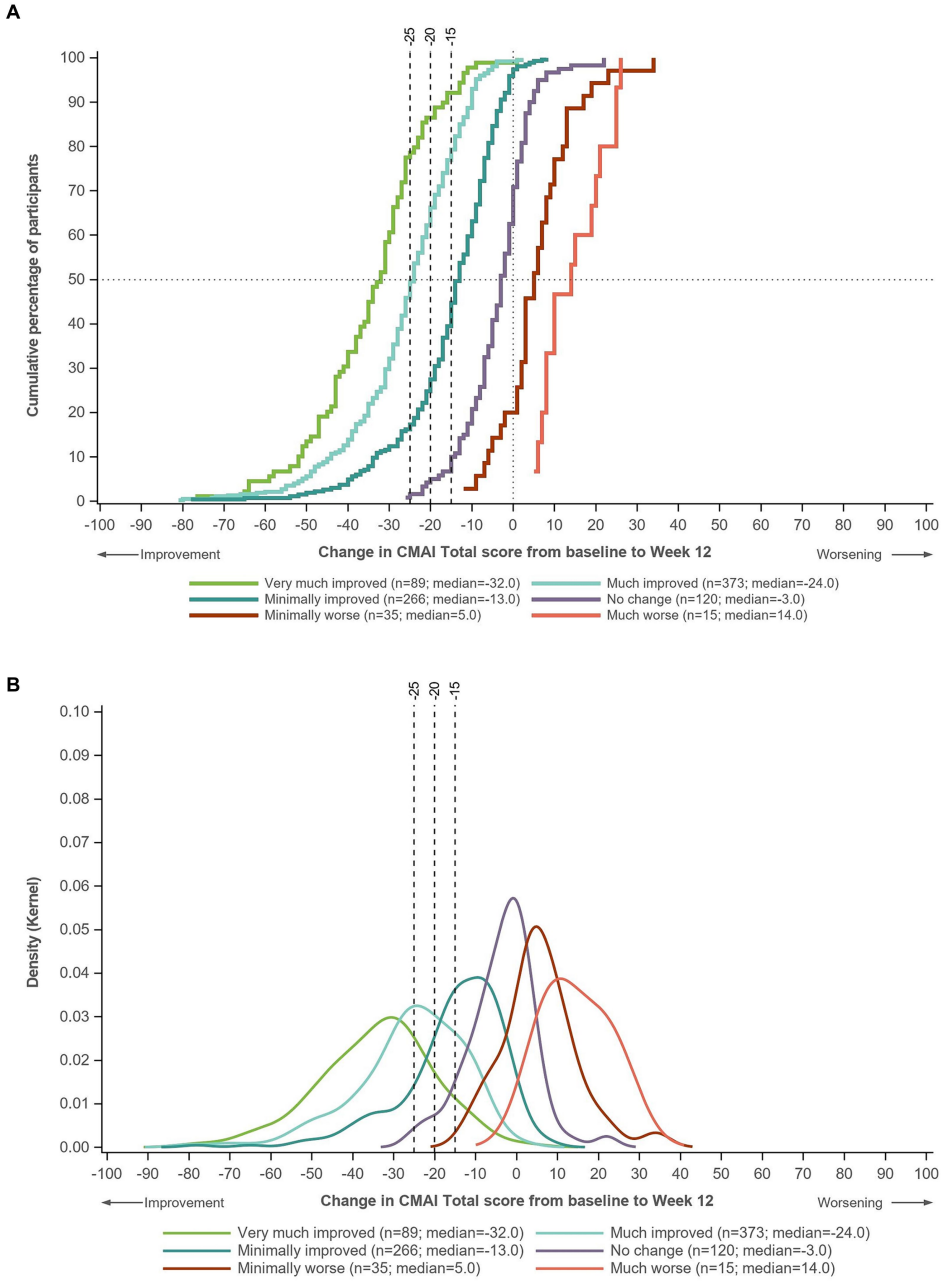
**(A)** eCDF and **(B)** PDF of change in CMAI Total score in groups defined by CGI-I score at Week 12. Data for Trials 1–3 pooled. Very much improved, CGI-I score of 1; much improved, CGI-I score of 2; minimally improved, CGI-I score of 3; no change, CGI-I score of 4; minimally worse, CGI-I score of 5; much worse, CGI-I score of 6. CGI-I, Clinical Global Impression-Improvement; CMAI, Cohen-Mansfield Agitation Inventory; eCDF, empirical cumulative distribution function; PDF, probability density function.

Using pooled data from all three trials, eCDFs and PDFs showed good distribution and separation of the change in CMAI Total score between the categories defined by CGI-S score change (Figure 1) and CGI-I score (Figure 2).

On the eCDF by CGI-S score change, ~80% of patients in the moderate improvement group and ~ 60% of patients in the small improvement group had a CMAI Total score change of −15 points from baseline to Week 12, compared with <20% of patients in the stable group (Figure 1A). Approximately 70% of patients in the moderate improvement group and ~ 40% in the small improvement group had a CMAI Total score change of −20 points from baseline to Week 12, compared with ~10% in the stable group. Approximately 60% of patients in the moderate improvement group had a CMAI Total score change of −25 points from baseline to Week 12, compared with <30% in the small improvement group and ~ 5% in the stable group. Similar distributions were observed on the eCDF by CGI-I score (Figure 2A).

### 3.5 Distribution-based analyses

The MDCs for CMAI Total score were 11.4 points using pooled data from Trials 1 and 2, 10.5 points for Trial 3, and 11.8 points using pooled data from all three trials. These MDCs were generally lower than the anchor-suggested thresholds, supporting that the CMAI has sufficient precision to estimate a threshold of this magnitude (Table 4).

**Table 4:** Distribution-based change statistics for CMAI Total score at baseline.

Trial	SD_BL_	½ SD_BL_	SEM^a^	MDC^b^
Trials 1 and 2 pooled	16.8	8.4	4.1	11.4
Trial 3	16.9	8.5	3.8	10.5
Trials 1–3 pooled	17.4	8.7	4.3	11.8

^a^SEM = SD_BL_ × √(1 − *r*), where *r* is the reliability coefficient estimated by ICC in stable patients defined as “no change” in CGI-I at Week 2. ^b^MDC = 1.96 × √2 × SEM. CGI-I, Clinical Global impression–Improvement; CMAI, Cohen-Mansfield Agitation Inventory; ICC, intra-class correlation coefficient; MDC, minimum detectable change; SD_BL_, standard deviation at baseline; SEM, standard error of measurement.

### 3.6 Triangulation of a meaningful within-patient change threshold

Based on triangulation of evidence from the anchor- and distribution-based analyses of the pooled data from Trials 1 and 2, an MWPC threshold for CMAI Total score in the range of −15 to −20 points was supported, with CGI-S results suggesting slightly larger values. As the FDA guidance favors global severity scales as an anchor (29), the more conservative threshold of −20 points was selected.

The analyses for Trial 3 supported this threshold range; however, a higher threshold (around −25 points) was needed to cover the larger improvement comparisons. Overall, the analyses of three trials supported an MWPC threshold for CMAI Total score of −20 points, with a threshold range of −15 to −25 points.

### 3.7 Brexpiprazole responder analyses using the meaningful within-patient change threshold

**Figure 3:**
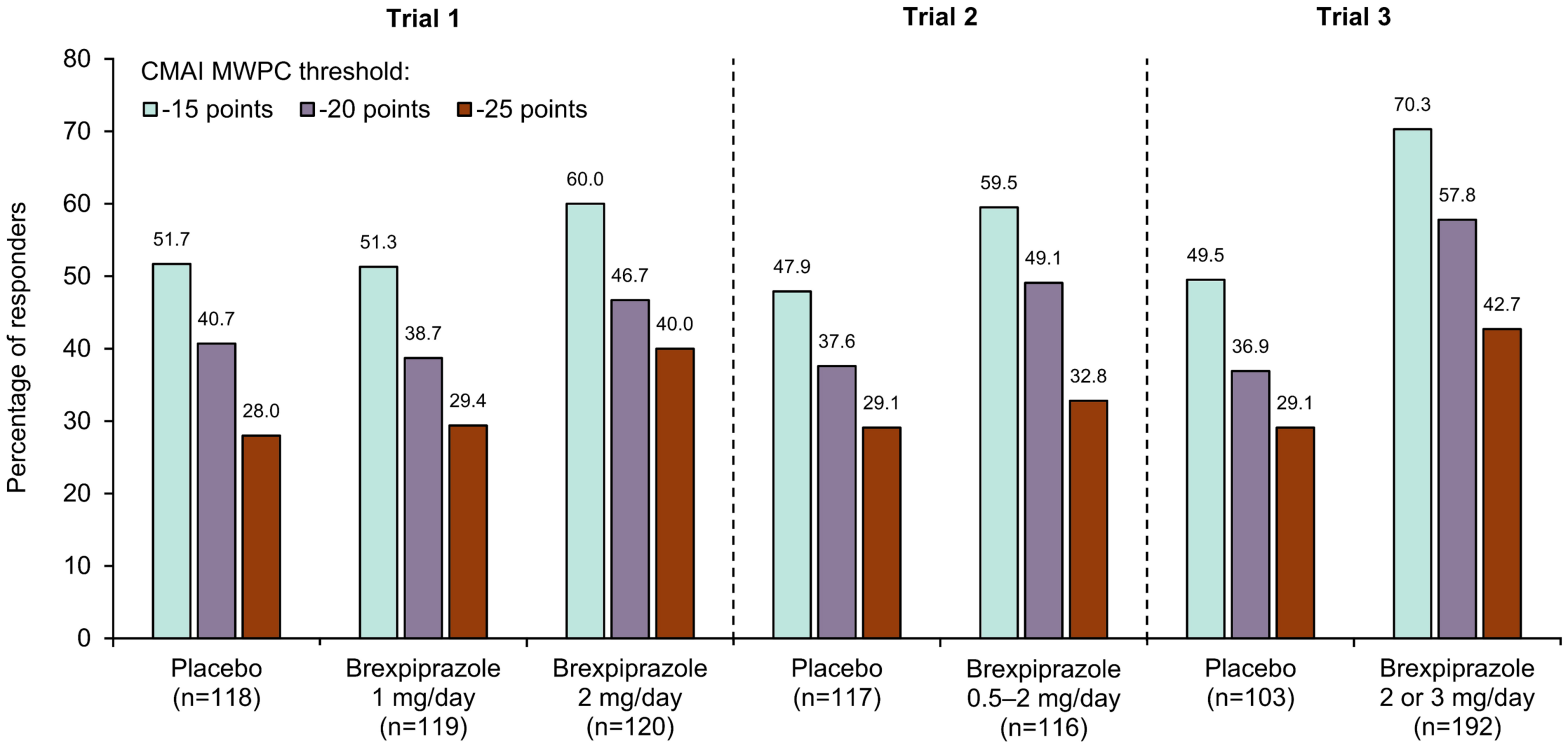
Response rates at Week 12 for CMAI Total score at different MWPC thresholds in brexpiprazole clinical trials. CMAI, Cohen-Mansfield Agitation Inventory; MWPC, meaningful within-patient change.

Responder analyses were conducted using CMAI Total score MWPC threshold values of −15, −20, and − 25 points. The proportion of responders at Week 12 was higher for brexpiprazole 2 or 3 mg/day than for placebo at all three response thresholds (Figure 3). For each treatment arm, the percentage of responders decreased with an increase in threshold value.

In Trial 1, the separation between the brexpiprazole 2 mg and placebo groups was greatest with the −25-point threshold. In Trials 2 and 3, the separation between the brexpiprazole and placebo groups was greatest with the −15-point and − 20-point thresholds.

## 4 Discussion

Defining an MWPC threshold helps with the interpretation of the clinical relevance of clinical trial results (29, 34). An MWPC represents the magnitude of change that can be considered clinically relevant at the individual-patient level (35). This differs from the minimal clinically important difference (MCID), which represents a clinically relevant change at the group-means level, and which is useful to calculate sample size in clinical trials (35). MWPCs can be used in responder analyses to identify specific patients who had a meaningful response to treatment, which can complement analyses of group-mean differences and statistical significance (35).

Assessment of meaningful improvement of agitation is central to developing new treatments for agitation in Alzheimer’s disease. It is of clinical benefit to determine an MWPC threshold for the CMAI because this scale has been used in clinical trials to assess agitation in dementia, including the trials that were submitted to the FDA for the approval of brexpiprazole for the treatment of agitation associated with dementia due to Alzheimer’s disease. Using data from the brexpiprazole Phase 3 clinical program, triangulation of various analyses support an MWPC threshold of −20 points for CMAI Total score, with a threshold range of −15 to −25 points. This was a rigorous analysis, using multiple techniques and two anchor scales, as recommended by the FDA (29). Use of multiple approaches to evaluate an MWPC is important, since different measures of meaningful change may resonate with different decision makers. For example, anchor-based methods are generally preferred for regulatory discussions, with distribution-based methods considered supportive (29, 35). The present CMAI Total score threshold of −20 points approximately corresponds to a two-point improvement in CGI-S score and a score of 2 on the CGI-I. Patients with moderate-to-marked agitation who have reductions in CMAI Total score of ≥20 points can therefore be considered as responding to treatment in a clinically meaningful manner.

Prior analysis of data from an observational study of the course of agitation in patients with Alzheimer’s disease (the “A3C” study) found an MWPC of −17 points for CMAI Total score changes over 3 months (whereas the study used the term “MCID”, the methods suggest it was an MWPC) (36). Although the A3C study used a different anchor and different methodology to the present study, the patient samples were similar, and it is reassuring that the reported MWPC of −17 points is consistent with the presently determined threshold range of −15 to −25 points.

In the United States, the approved target dose of brexpiprazole for the treatment of agitation associated with dementia due to Alzheimer’s disease is 2 mg/day, which may be increased to a maximum of 3 mg/day (21). At these doses, responder analyses based on the −20-point threshold were able to distinguish active treatment from placebo, with a greater percentage of patients treated with brexpiprazole achieving meaningful improvements in CMAI Total score than those who received placebo. Response rates based on the −20-point threshold were higher in Trial 3 than in Trials 1 and 2, which may reflect the enriched sample for agitation in Trial 3.

Meaningful within-patient change thresholds are specific to the patient population studied (35). The present analysis is limited by the trial inclusion criteria, which means that results may not be generalizable to all patients with agitation associated with dementia due to Alzheimer’s disease. Furthermore, although the trials were conducted across two continents, over 95% of the patient sample were White, which may limit the generalizability of the MWPC threshold to other races. The trial is also limited in that both of the anchor scales were clinician rated, meaning that the patient and caregiver perspectives were not considered.

In conclusion, based on anchor- and distribution-based methods, MWPC analyses support an improvement threshold of −20 points for CMAI Total score in patients with agitation associated with dementia due to Alzheimer’s disease, with a threshold range of −15 to −25 points. Using this threshold, a greater proportion of patients on brexpiprazole 2 or 3 mg/day than placebo achieved a meaningful improvement in agitation in the brexpiprazole Phase 3 clinical program.

## Data availability statement

To submit inquiries related to Otsuka clinical research, or to request access to individual participant data (IPD) associated with any Otsuka clinical trial, please visit: https://clinical-trials.otsuka.com/. For all approved IPD access requests, Otsuka will share anonymized IPD on a remotely accessible data sharing platform.

## Ethics statement

Ethical approval was not required for the studies involving humans because the study involved retrospective analysis of data from clinical trials with ethical approval. All patients and/or their legal representatives provided written or electronic informed consent prior to the start of each trial.

## Author contributions

JM: Conceptualization, Data curation, Formal analysis, Investigation, Methodology, Resources, Software, Supervision, Validation, Visualization, Writing – review & editing. KC: Data curation, Formal analysis, Investigation, Methodology, Software, Validation, Visualization, Writing – review & editing. AL: Data curation, Formal analysis, Investigation, Methodology, Software, Validation, Visualization, Writing – review & editing. KGL: Conceptualization, Investigation, Methodology, Writing – review & editing. JA: Project administration, Writing – review & editing. NH: Conceptualization, Writing – review & editing. DO: Conceptualization, Funding acquisition, Investigation, Methodology, Project administration, Resources, Supervision, Writing – review & editing.
